# Reducing metal artifacts by restricting negative pixels

**DOI:** 10.1186/s42492-021-00083-z

**Published:** 2021-06-01

**Authors:** Gengsheng L. Zeng, Megan Zeng

**Affiliations:** 1 Department of Computer Science, Utah Valley University, Orem, UT 84058 USA; 2 Department of Radiology and Imaging Sciences, University of Utah, Salt Lake City, UT 84108 USA; 3 Department of Electrical Engineering and Computer Science, University of California at Berkeley, Berkeley, CA 94720 USA

**Keywords:** Filtered backprojection image reconstruction, Iterative algorithm, Metal artifact reduction, Objective function, X-ray computed tomography

## Abstract

When the object contains metals, its x-ray computed tomography (CT) images are normally affected by streaking artifacts. These artifacts are mainly caused by the x-ray beam hardening effects, which deviate the measurements from their true values. One interesting observation of the metal artifacts is that certain regions of the metal artifacts often appear as negative pixel values. Our novel idea in this paper is to set up an objective function that restricts the negative pixel values in the image. We must point out that the naïve idea of setting the negative pixel values in the reconstructed image to zero does not give the same result. This paper proposes an iterative algorithm to optimize this objective function, and the unknowns are the metal affected projections. Once the metal affected projections are estimated, the filtered backprojection algorithm is used to reconstruct the final image. This paper applies the proposed algorithm to some airport bag CT scans. The bags all contain unknown metallic objects. The metal artifacts are effectively reduced by the proposed algorithm.

## Introduction

Due to the wide energy spectrum of x-rays, beam hardening effects are severe when the object being imaged contains metals. The beam hardening effects introduce large errors in the x-ray computed tomography (CT) projection measurements. These measurement errors in turn produce artifacts in the reconstructed CT images. Typical metal artifacts appear as dark and bright streakings. This metal artifact problem has been recognized for a long time and it is still an open problem [1].

Most methods to combat the metal artifacts are iterative algorithm based [2–9]. Among these iterative algorithms, projection data inpainting is popular. The basic priciple of inpainting is first to remove the metal affected measurements and to assume that there is no metal in the object. Next, estimation methods such as interpolation, lowpass filtration, or some non-linear approaches are used to inpaint the measurements that are artifacially removed in the first step. Till now, the impainting methods are still not accurate enough to reproduce the true metal-free projections.

The modern metal artifact reduction methods are iterative methods. Iterative algorithms are designed to optimize an objective function, which can contain Bayesian terms. For example, the total variation (TV) norm is effective in enforcing the peicewise constant prior [10, 11]. Noise weighting is often incorporated in the objective function as well.

Our proposed method is inspired by the observation that the metal artifacts usually have regions with negative pixel values. The innovation of this paper is to establish an objective function that restricts the negative pixel values in the reconstructed images. The proposed method will be presented in the next section. Results with real x-ray CT measurements are presented. The measurements are obtained from airport bags that contain metal objects inside.

## Methods

A usual objective function in image reconstruction consists of two parts: the data fidelity part and the Bayesian part. The data fidelity part projects the image array to generate pseudo projections and then matches them to the measurements. Noise weighting can be applied in the data fidelity part. The main purpose of the Bayesian part is for regularization because the image reconstruction problem may be ill posed. An L_2_-norm of the reconstructed image can be used to regularize the image to enforce smoothness. The TV norm of the image can be used to denoise and maintain the sharp edges, by encouraging the piecewise constant constraint. Projection data inpainting is usually required before iterative image reconstruction. Unfortunately, inpainting methods are problematic and the pseudo projections are not the same as the projections when metals are absent.

Our innovation is an objective function that does not have a data fidelity term. Our objective function is inspired by the observation that the metal artifacts often have regions with negative pixel values. However, the x-ray attenuation coefficients cannot be negative. This paper proposes an objective function, which is the squared L_2_-norm of the negative pixel values of the filtered backprojection (FBP) reconstruction.

Let *A* be the operator of the FBP algorithm, *P* be the projection measurements, and *X* be the FBP reconstruction. Both *P* and *X* are expressed in the vector form, and *A* is expressed in the matrix form. The FBP reconstruction *X* is *AP*. The elements in *X* are *x*_*i*_. Let *Y* be the column vector containing the entries

1 $$ {y}_i=\min \left(0,{x}_i\right) $$

Thus, the vector *Y* is the same as the FBP reconstruction *X*, except that all positive pixels of *X* are set to zero. The proposed objective function is the squared L_2_-norm of *Y* as

2 $$ F={\left\Vert Y\right\Vert}_2^2 $$

We would like to minimize this objective function (2). The variables for this objective function are the metal affected projections *P*_*M*_. Here, the entries in *P*_*M*_ are determined by following procedure:

- Step 1: Use the FBP algorithm to generate a raw image *X*_*raw*_ using raw projection measurements *P*. The raw image may contain severe metal artifacts.
- Step 2: Segment the raw image to obtain a metal-only image, using a threshold value, for example, as the 1/3 of the maximum image value of *X*_*raw*_. All image values smaller than this threshold value are set to zero.
- Step 3: Forward project the metal-only image to obtain the indices of *P*_*M*_. Other projections in *P* are not affected by metal and are denoted by *P*_*notM*_.

We propose to use a gradient descent algorithm to minimize the objective function (2) by updating the variables in *P*_*M*_. Let *p*_*j*_ be an entry in *P*_*M*_. To find the gradient of *∂F*/*∂p*_*j*_ is not straightforward, because the min function in (1) makes (1) undifferentiable. We can use the subdifferential concept to find the gradient of *∂F*/*∂p*_*j*_ as [12, 13].

3 $$ \nabla F=2{A}^T\min \left\{0, AP\right\}=2{A}^TY $$

where *A*^*T*^ is the adjoint operator of the FBP algorithm and min{0, *AP*} sets each positive entry of *AP* to zero. Here, *AP* is the FBP image reconstruction using projections *P*, and *A*^*T*^ is the forward projection followed by the ramp filtration with the one-dimensional convolution kernel, which is defined as

4 $$ h(n)=\left\{\begin{array}{ccc}\frac{1}{4}& if& n=0,\\ {}-\frac{1}{{\left( n\pi \right)}^2}& if& n\  is\  odd,\\ {}0& otherwise.& \end{array}\right. $$

The gradient descent iterative algorithm is given as

5 $$ {P}_M^{\left(k+1\right)}={P}_M^{(k)}-\beta D{A}^T\min \left\{0,A{P}^{(k)}\right\} $$

where the super script (*k*) is the iteration index. The projection vector *P* consists of two parts: the metal affected part *P*_*M*_ and the metal not affected part *P*_*notM*_. The metal not affected part *P*_*notM*_ does not get updated from iteration to iteration. The operator *D* in (5) is a dimension reduction operator that discards the entries in *P*_*notM*_. The parameter *β* in (5) controls the step size of the gradient descent algorithm.

The proposed algorithm (5) was implemented in MATLAB and applied to some CT data of airport bags. The original projections of airport bags were acquired with an Imatron C300 clinical CT scanner. The contents and details were not disclosed to us. The detector and x-ray source details were unknown. The objects were treated as unknown objects.

The step size *β* was chosen to be 1, and the number of iteration was 500. The original CT data resolution was 0.5 mm. The original cone-beam data was reformatted into parallel-beam, lower-resolution data with 0.92 mm spatial resolution in this paper. The number of views for the scaled-down version was 180 over 180°. The field-of-view was 475 mm. The image was 475 mm × 475 mm. The parallel-beam data had 597 bins on each detection row, and the bin size was 0.92 mm. The reconstructed image was in a 420 × 420 two-dimensional array and the pixel size was 0.92 mm.

In our airport bag application, the ground truth is unavailable. Therefore, quantitative evaluation is not appropriated. For metal reduction evaluations, ref. [14] suggested task-based human observer studies or channelized Hotelling observer studies. The task is usually small lesion detection in medical imaging, and the ground truth should be known. Therefore, the suggested studies do not apply in our situation. The only evaluation we can perform is visual appearance evaluation, which is subjective and may not be reliable enough to make any definite conclusions. In this paper, we are very careful not to make any strong claims about the superiority of the proposed algorithm. The only claim we make in this paper is that the proposed method is different from the method that sets all negative image pixels to zeros.

## Results

**Fig. 1:**
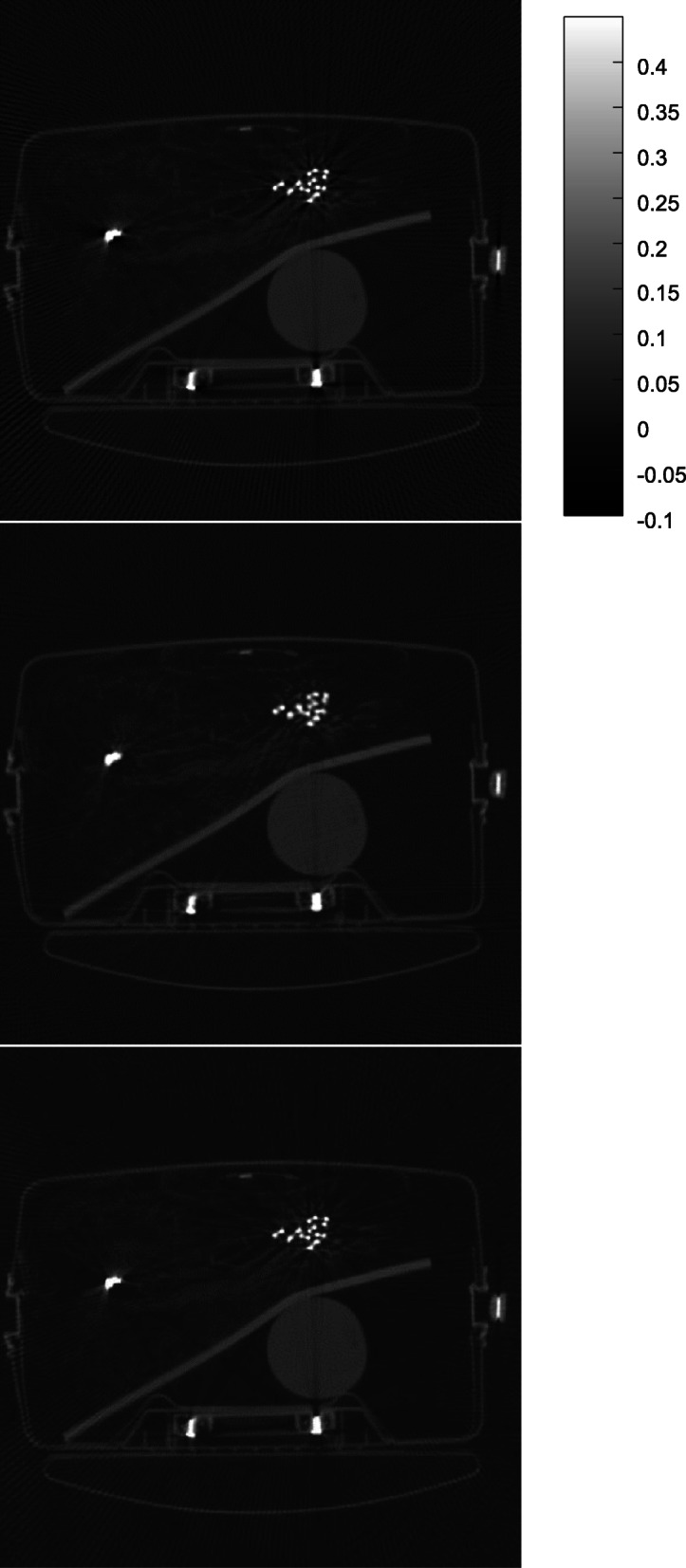
CT image reconstruction of airport bag #1. Top: the raw FBP reconstruction; Middle: the reconstruction using the proposed algorithm; Bottom: the raw FBP reconstruction with all negative pixel values replaced by zeros

**Fig. 2:**
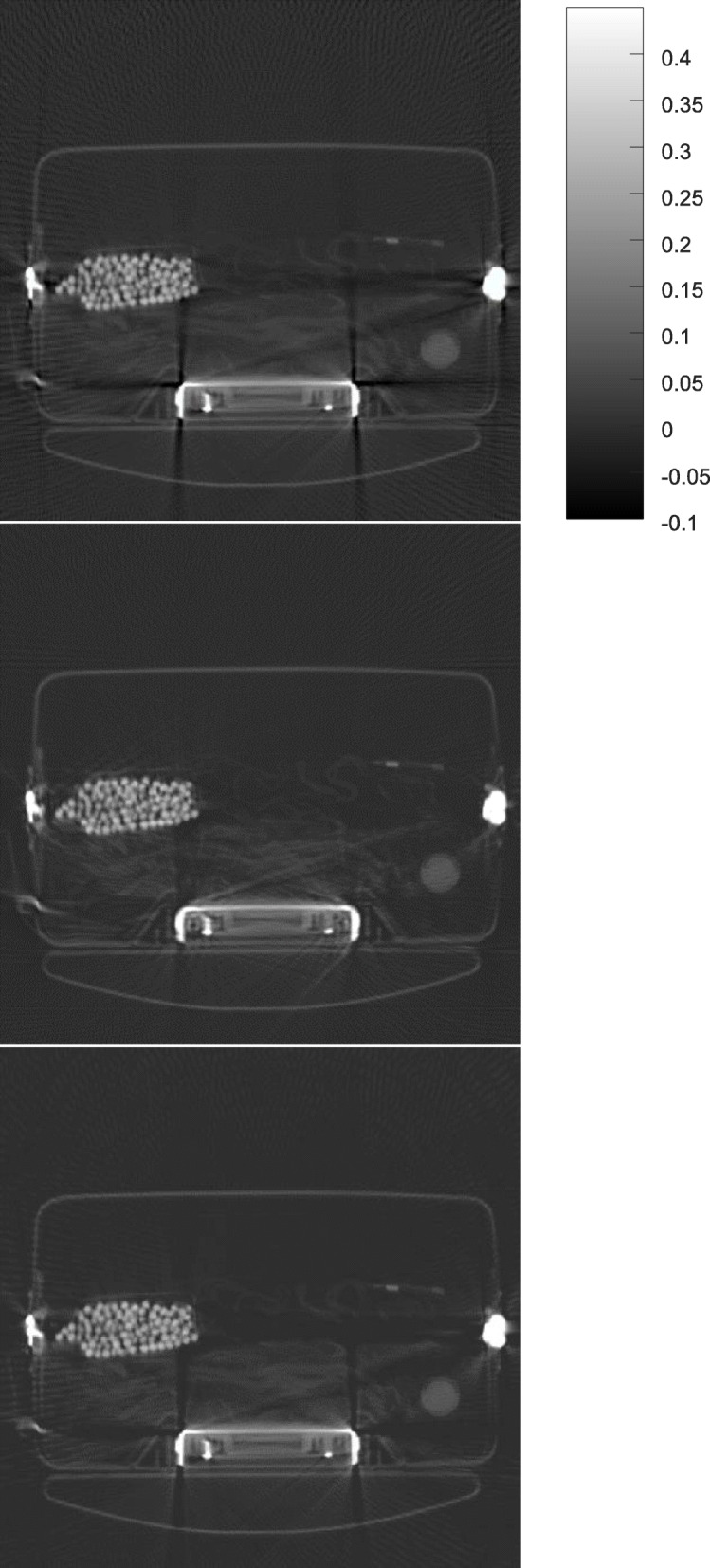
CT image reconstruction of airport bag #2. Top: the raw FBP reconstruction; Middle: the reconstruction using the proposed algorithm; Bottom: the raw FBP reconstruction with all negative pixel values replaced by zeros

**Fig. 3:**
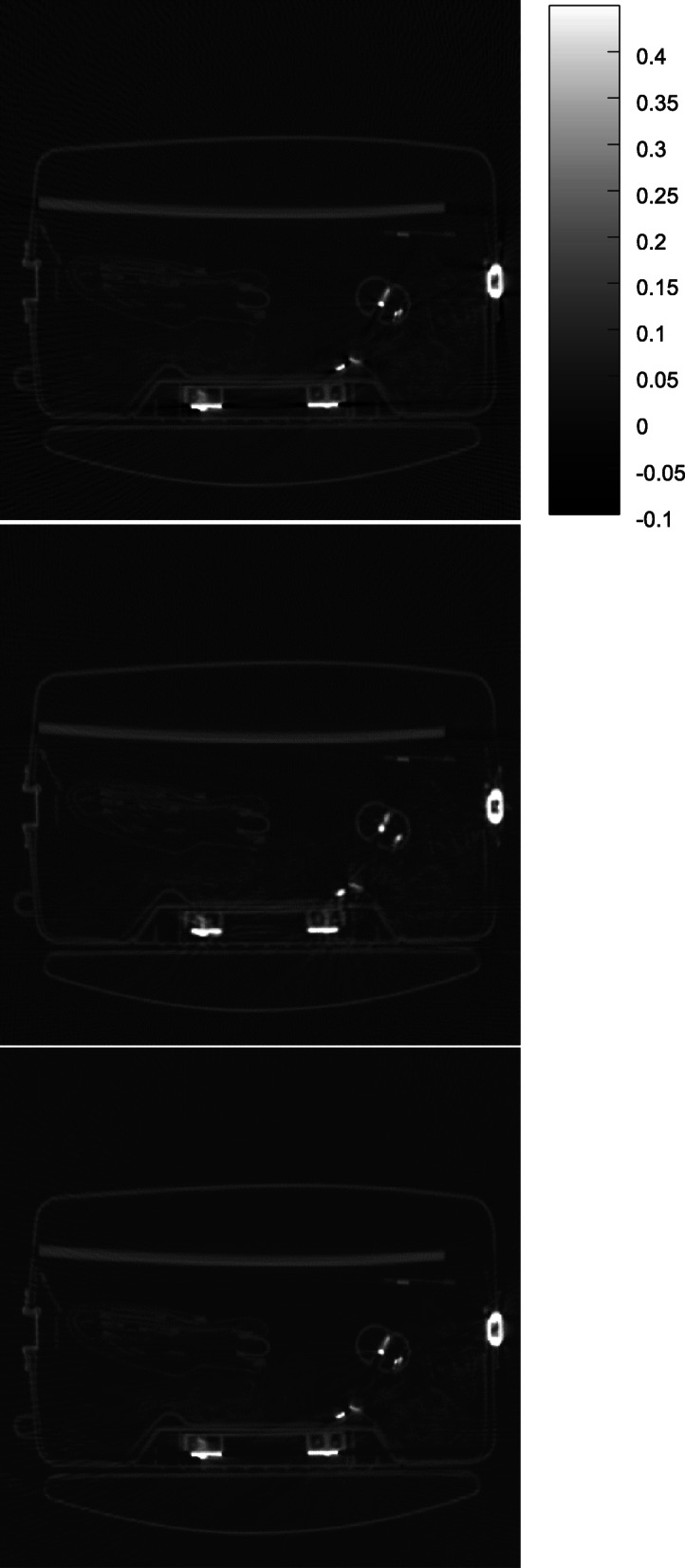
CT image reconstruction of airport bag #3. Top: the raw FBP reconstruction; Middle: the reconstruction using the proposed algorithm; Bottom: the raw FBP reconstruction with all negative pixel values replaced by zeros

**Fig. 4:**
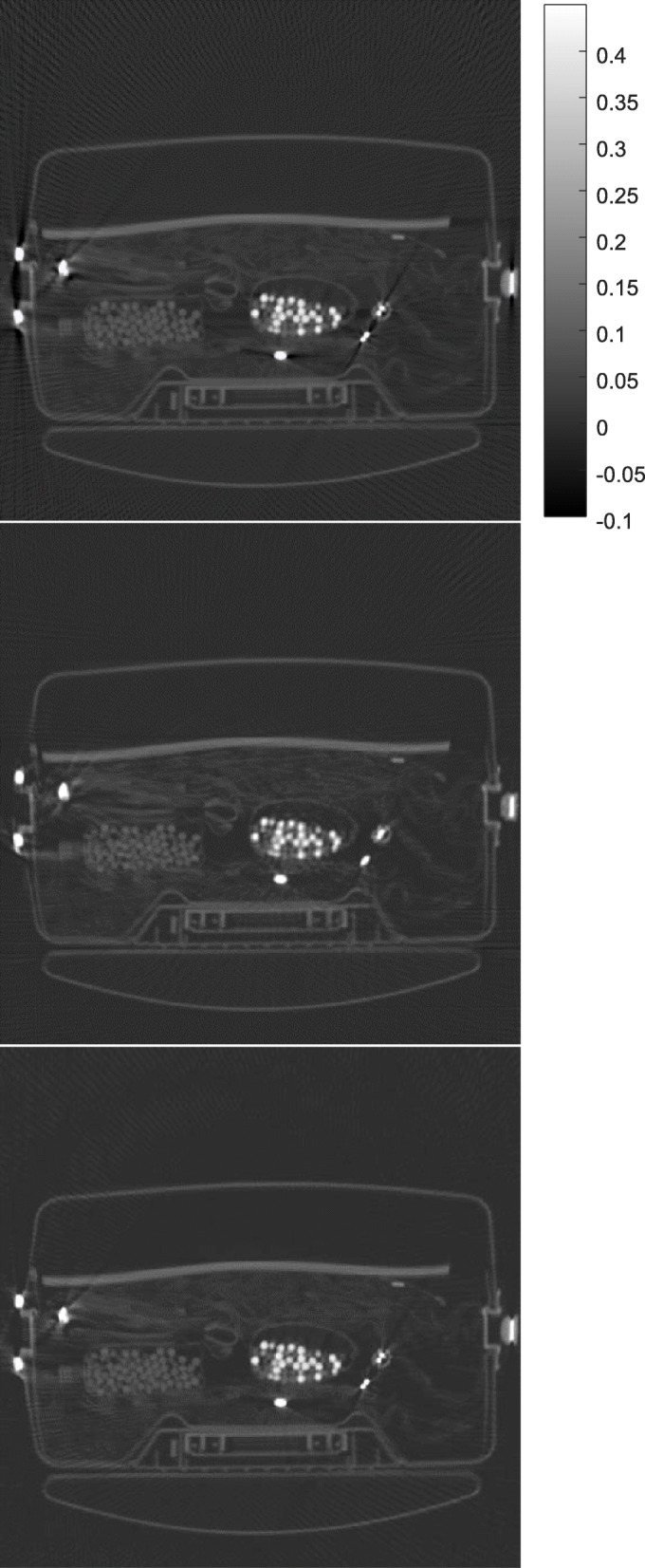
CT image reconstruction of airport bag #4. Top: the raw FBP reconstruction; Middle: the reconstruction using the proposed algorithm; Bottom: the raw FBP reconstruction with all negative pixel values replaced by zeros

**Fig. 5:**
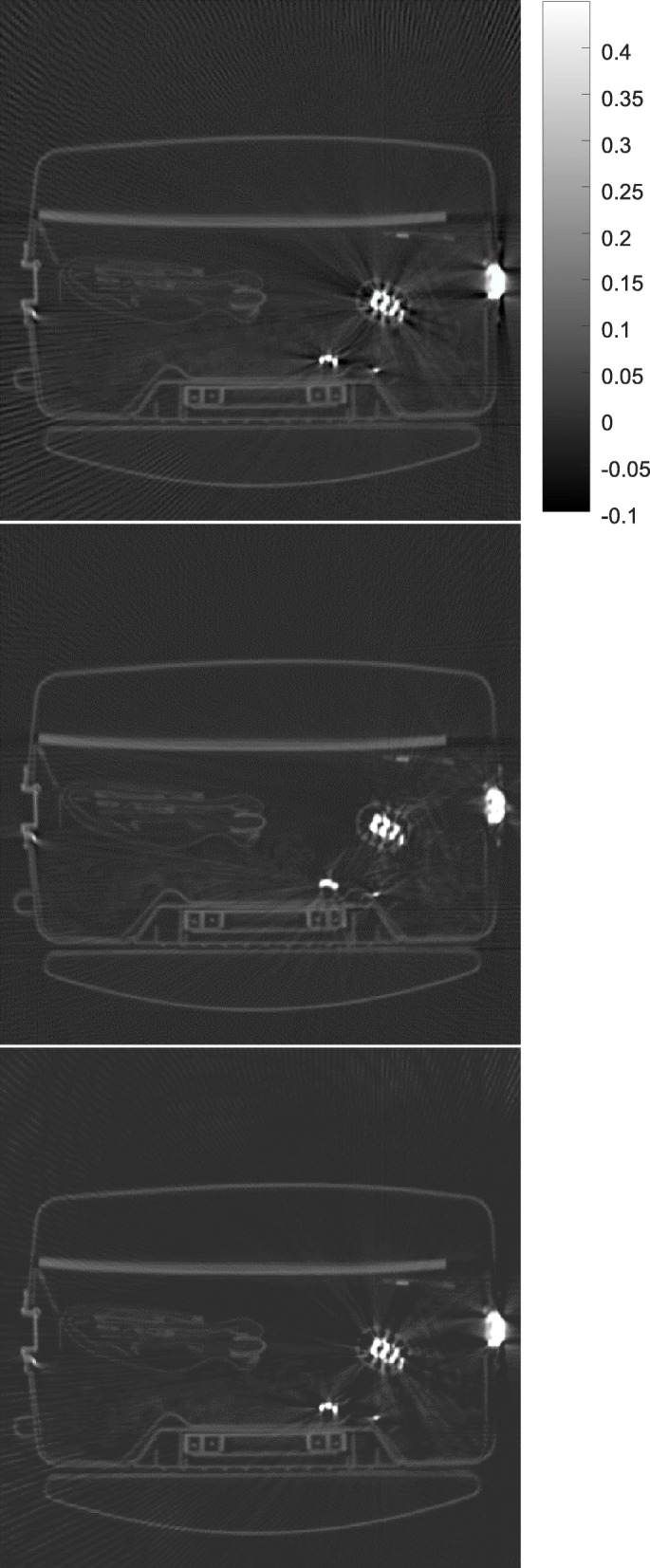
CT image reconstruction of airport bag #5. Top: the raw FBP reconstruction; Middle: the reconstruction using the proposed algorithm; Bottom: the raw FBP reconstruction with all negative pixel values replaced by zeros

Some results obtained by the proposed algorithm are shown in Figs. 1, 2, 3, 4 and 5 for 5 different airport bags, respectively. Three images are shown in each figure: the raw FBP reconstruction, the proposed algorithm followed by the FBP reconstruction, and the raw FBP reconstruction with negative pixels replaced by zeros. The negative values are shown as the darkest color. The metals appear as the brightest color. Since the attenuation coeffecients are mach greater than the rest of the object, the display window is set to [− 0.1*a*, 0.45*a*], where *a* is the maximum image value. The display window for the raw image and the final image is the same.

The negative image pixel values are only appear in the close neighborhood of the metals in the raw FBP reconstructions. After the proposed iterative algorithm removes the negative image pixels, the dark streaking artifacts are also reduced. This phenomenon cannot be achieved by simply setting the negative image pixel values to zeros in the raw FBP reconstructions.

The raw FBP reconstruction images for bags 1–5 with the negative image pixel values replaced by zeros are also displayed in Figs. 1, 2, 3, 4 and 5 for comparison purpose and they look almost the same as the raw FBP images.

**Fig. 6:**
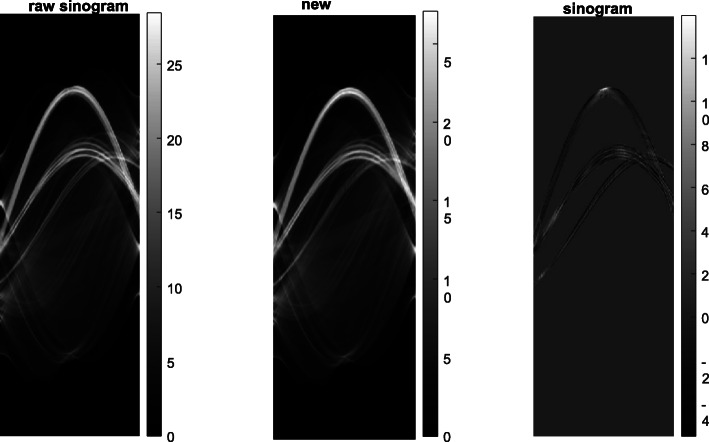
Sinograms for airport bag #5. Left: the raw sinogram; Middle: the processed sinogram using the proposed algorithm; Right: the difference between the processed sinogram and the raw sinogram

The raw sinogram and processed sinogram are compared in Fig. 6. The proposed algorithm does not alter the sinogram values if they are not affected by the metals.

As a side product, the angularly aliasing artifacts (due to insuffecient view angles) are also reduced with the proposed algorithm.

## Discussion

This paper uses a unique objective function for reducing the errors in the projection measurements. The errors are caused by beam hardening effects of the metalic objects. The establishment of the objective function is inspired by the observation that the metal artifacts in CT FBP reconstructions may have some negative undershoots close to the metals. The new objective function is to penalize those negative valued pixels. It is interesting to observe that once the negative undershoots are removed, the streaking artifacts are significantly reduced, even though the streaking artifacts may not contain negative pixels.

The traditional iterative algorithm’s main goal is to iteratively reconstruct the image. On the other hand, we use the FBP algorithm to reconstruct the image in every iteration of the proposed algorithm. Most iterative algorithms use image pixels as the unknowns, while the proposed algorithm uses the metal affected projections as the unknowns.

From our knowledge, this is the first time in image reconstruction that the L_2_-norm of the negative pixels is used as the objective function to be minimized.

It is not straightforward to optimize an objective function that is undifferentiable. We do not know the partial derivatives of the objective function with respect to the varaibles, which are the metal affected projections. In this paper, we propose a subdifferential to approximate gradient, which does not exist. With this subdifferential, a gradient descent algorithm is developed and tested with real CT data.

From another point of view, the proposed algorithm is able to minimize the some features of the metal artifacts. The phenomenon of negative undershoots is one of the metal artifact features. There could be other features. In principle, once we can express the features, we are able to minimize them. In our previous paper, the TV was used as a feature for the metal artiacts [15]. The TV norm is useful and effective, but it may smooth the image too much.

We would like to point out that our method does not belong to the traditional category of projection data inpainting. In traditional projection data inpainting, the metalic objects are first removed from the image by segmentation methods, and the corresponding metal affected projections are removed as well. The projection data inpainting methods then replace the removed projections by estimations from the neighbors. The metal-free image is reconstructed from the newly modifided projections. The metal-only image and the metal-free image are combined to generate the final image. In our proposed algorihtm, the metalic objects and their projections are never removed. We don’t reconstruct metal-free and metal-only images separately. The proposed algorithm overcomes some difficulties of performing data inpainting.

## Conclusions

This paper suggests that the total ‘energy’ of the negative image pixels be used as a feature of the metal artifacts. Minimizing this ‘energy’ leads to minimizing metal artifacts. Real airport CT scans were used to verify the feasibility of the proposed algorithm. The results indicate that the dark streaking artifacts around the metallic objects have been reduced. The images produced by the proposed algorithm are different from the raw FBP reconstruction images with the negative image pixel values replaced by zeros.

## Data Availability

Not applicable.

## Abbreviations

CT: Computed tomography
FBP: Filtered backprojection
TV: Total variation
